# The effect of business cycle expectations on the German apprenticeship market: estimating the impact of Covid-19

**DOI:** 10.1186/s40461-020-00094-9

**Published:** 2020-09-18

**Authors:** Samuel Muehlemann, Harald Pfeifer, Bernhard H. Wittek

**Affiliations:** 1 LMU Munich, Munich School of Management, Geschwister-Scholl-Platz 1, 80539 Munich, Germany; 2 IZA Institute of Labor Economics, Bonn, Germany; 3 Federal Institute for Vocational Education & Training (BIBB), Robert-Schuman-Platz 3, 53175 Bonn, Germany; 4 Research Centre for Education and the Labour Market (ROA), School of Business and Economics, Maastricht University, Tongersestraat 53, 6200 MD Maastricht, Netherlands

**Keywords:** Apprenticeship market, Covid-19, Coronavirus, Business cycle expectations, J23, J24, M53

## Abstract

A firm’s expectation about the future business cycle is an important determinant of the decision to train apprentices, especially as German firms typically offer apprenticeships to either fill future skilled worker positions, or as a substitute for other types of labor. The current coronavirus crisis will have a strong and negative impact on the German economy, according to the current business cycle expectations of German firms. To the extent that the training decisions of firms depend on these perceptions, we expect a downward shift in firm demand for apprentices and consequently also a decrease in the equilibrium number of apprenticeship contracts. To assess the impact of changes in business cycle expectations, we analyze German data on the apprenticeship market at the state-level and at the occupation-level within states from 2007 to 2019. We apply first-differences regressions to account for unobserved heterogeneity across states and occupations, allowing us to identify the association between changes in two popular measures of business cycle expectations (the ifo Business Climate Index and the ifo Employment Barometer) and subsequent changes in the demand for apprentices, the number of new apprenticeship contracts, unfilled vacancies and unsuccessful applicants. We find that the German apprenticeship market prior to the current crisis can be characterized by excess demand for apprentices (although there are matching problems in some states, with both a high share of unfilled vacancies and a high share of unsuccessful applicants). Taking into account the most recent data on business cycle expectations up to June 2020, we estimate that the coronavirus-related decrease in firms’ expectations about the business cycle can be associated with a predicted 8% decrease in firm demand for apprentices and a 6% decrease in the number of new apprenticeship positions in Germany compared to 2019 (− 30,000 apprenticeship contracts; 95% confidence interval: ± 8000).

## Introduction

The Covid-19 pandemic is fundamentally altering outlooks in many parts of society. With the immediate health implications playing out across the globe, the economic impact is starting to show in labor markets as well. The United States has seen an unprecedented jump in unemployment figures to 23.1 million in April 2020, recovering slightly to 17.8 million by June, up from 7.1 million three months before (USBLS 2020b).^1^ While Germany has not experienced comparably drastic developments so far, unemployment numbers rose from 2.3 million in March to 2.9 million in June (Federal Employment Agency 2020a), economic expectations about the near future, here too, have turned decisively pessimistic. The effects of the expected downturn are likely to be felt differently across industries and segments of the population. In past crises, especially the young have suffered (see, e.g., Bell and Blanchflower 2011), albeit with marked differences between countries. In 2010, as much of the world was dealing with what has been termed the *Great Recession*, the unemployment rate for the youngest labor market cohort (15–24 year-olds) mounted to 41.6% in Spain, but was only at 9.9% in Germany (European Commission 2012). As researchers and policy makers seek explanations for such stark disparities, the use of apprenticeship systems, with a significant amount of on-the-job training, has frequently moved to the front. To what extent are such programs subject to the ups and downs of the economy and can they add to the resilience of a labor market during crises, for example, by facilitating the transition from school to full-time employment? To answer such questions, we direct our attention towards the impact of business cycle developments on the demand for apprentices. If such vocational education and training (VET) programs appear more robust to economic downturns, they may plausibly play a larger role in supporting the labor market as a whole. Thus far, research has outlined theoretical possibilities for both pro-cyclical effects, largely driven by reduced staffing needs during an economic downturn, as well as counter-cyclical outcomes, caused, for example, by lowered opportunity costs of training during less productive periods. Most empirical studies to date suggest a pro-cyclical, but small to moderate effect (see, e.g., Mühlemann et al. 2009). Furthermore, what matters in this regard, especially during more pronounced economic crises, seems to be more the longer term outlook, not the specific effect a downturn has on an individual company (Bellmann et al. 2014). We seek to build on these prior findings and investigate how company expectations about business cycle developments influence apprenticeship provision. Subsequently, we attempt to project the likely short-term impact of Covid-19 on the German apprenticeship system, based on currently available information. We employ annual data on the number of firm-sponsored apprenticeship contracts between 2007 and 2018, processed by the Federal Statistical Office (DESTATIS) and provided by the Federal Institute for Vocational Education and Training (BIBB). To further differentiate the mechanisms of the expected labor market effect, we utilize additional data on the demand for apprentices (i.e., the sum of posted apprenticeship vacancies and unfilled positions) as well as the number of both successful and unsuccessful applicants for apprenticeship positions from the Federal Employment Agency (2007–2019). Expectations about business cycle developments are measured through the ifo Business Climate Index (BCI) and the ifo Employment Barometer (EB), two publicly available, monthly surveys among German firms, which provide particularly current and useful insights into company expectations about the business cycle (Sauer and Wohlrabe 2020; Wohlrabe 2018). In investigating business cycle effects on this particularly relevant portion of the labor market and deriving a first estimate of the exceptional impact of the current Covid-19-linked downturn, we join a highly timely debate (see Lüthi and Wolter 2020b; Maier 2020 for initial contributions), while there are still possibilities for policy makers and organizational stakeholders to react to what is unfolding.

The succeeding sections are organized as follows. We first provide a brief overview of the relevant attributes of the German apprenticeship system and the decisive role firms play therein. Subsequently, we offer a summary of the literature on business cycle effects on training provision. Chapters 4 and 5 follow with descriptive statistics and further details on our main variables, as well as the identification strategy used in our analysis. We then present our results and conclude with an outlook on the impact of current economic expectations on apprenticeship contracts this fall.

## The German apprenticeship system and its reliance on firms to offer training positions

VET in Germany is offered through a publicly regulated “dual” system. It is referred to as such, as knowledge and skill acquisition does not only take place in vocational schools, but predominantly through practically oriented training and on-the-job learning at companies. Programs typically kick off in August or September each year and take students through two to three-and-a-half year apprenticeships, which result in qualifications for nationally recognized occupations. The specifics are heavily shaped by government regulations as well as negotiations between trade unions and employer associations.^2^ Entry to this system is administered mainly through private contracts between apprentices and training firms. It is this decision of the firm to invest in training apprentices that is paramount for the aggregated amount of training provided in the economy as a whole, especially as young school graduates depend on further qualification to attain labor market access. We will discuss theoretical motivations for firms to offer training positions in greater detail below, but such motivations may be heavily affected by firms’ expectations about the future business cycle. Once apprenticeships have begun, contractual training agreements can in effect not be terminated prematurely, unless for extraordinary circumstances. Dismissal is significantly easier during the first one to four months of the apprenticeship, a period referred to as “probation”. Legal precedent has also long upheld that contracts can generally be cancelled without notice or justification prior to the commencement of the apprenticeship (Federal Labour Court 1987). Once the apprenticeship has progressed beyond these initial stages, however, the legal and institutional commitment of the firm to complete the full, multi-year training period is strong (Dustmann and Schönberg 2012). Apart from organizational details, the training contract also includes the wages paid throughout the training period. While apprenticeship wages are in principle bound to collective agreements, the German apprenticeship market has seen the introduction of a regulatory minimum wage at the beginning of 2020. The wage floor was initially set at EUR 515 per month and designed to subsequently rise gradually to EUR 620 in 2023 (monthly wage in the first year of the apprenticeship program; BMBF 2019).^3^ While contracts typically also contain an option to transition into regular employment, they formally end upon completion of the apprenticeship and publication of final examination results (BMBF 2018). Hiring decisions of private companies are thus essential, when it comes to how much training is actually provided to each cohort. We do not know when exactly those decisions are made, but, depending on the individual industry, recruitment cycles start as early as one year prior to the commencement of the apprenticeship program and go all the way through August or September (see, e.g., Azubiyo GmbH 2020).^4^ Therefore, current, shorter-term expectations about economic prospects can be assumed to still factor heavily into this year’s capacity considerations. This has principally been confirmed in previous empirical analysis as well (Dietrich and Gerner 2007).

## Relevant literature

Research has identified two relevant underlying motivations for companies to hire apprentices: productivity, i.e. reasons driven by today’s production requirements, or investment, i.e. reasons driven by tomorrow’s production requirements (Lüthi and Wolter 2020a; Merrilees 1983; Wolter and Ryan 2011). The production motive (Lindley 1975) looks at apprentices essentially as just another input factor in the production process, a substitute for other labor, albeit often a less effective one. There may still be some investment period necessary at the beginning of training, but firms hire apprentices predominantly because of the net benefit they incur through the productive contributions of apprentices relative to their wages. The more forward-looking investment variant (Stevens 1994) has its roots in Becker’s (1964) human capital theory and looks at apprentices mainly as future skilled workers. In this regard, firms are willing to incur net costs during the initial training phase, in order to benefit in the future, for example, from secured or cheaper access to skilled labor. Prior research contributions have established certain characteristics of the labor market, such as information asymmetries (Acemoglu and Pischke 1999), mobility costs and a resulting reluctance to relocate (Beckmann 2002; Harhoff and Kane 1997), or rigidities associated with trade unions and works councils (Dustmann and Schönberg 2009; Kriechel et al. 2014), which may allow companies to suppress wage levels around the provision of training and recover their investment. Cost–benefit analyses suggest that investment motives play a decisive role for German firms in their considerations around the provisions of apprenticeships, driven significantly by labor market regulations (Mühlemann et al. 2010). The investment motive, thus, helps to explain findings in empirical studies that have demonstrated a willingness by German firms to incur substantial net costs during vocational training programs (Dionisius et al. 2009).

Based on these motivations, the effect of business cycle developments on training provision remains ambiguous ex-ante. Naturally, an economic downturn may lead to fewer transactions, lowered productivity and, therefore, a decrease in demand for labor, including apprentices. Furthermore, as unemployment increases, so does the availability of skilled labor in the labor market, which may alter investment considerations and cause a pro-cyclical response of apprenticeships to business cycles. If, on the other hand, skilled workers can be replaced by apprentices as a means to lower costs, a counter-cyclical movement could also seem plausible. Moreover, increasing training efforts during an economic slump can represent a prudent strategy from an investment perspective, if opportunity costs for training tasks of skilled employees are lowered and potential rewards for increased productivity are high, once the economy picks up again (Brunello 2009). Additionally, exit options may be relatively poor for apprentices during a recession, which may lower the perceived risks around a firm’s training investments (Bellmann et al. 2014). Likely, the temporal dimension of the expected downturn plays a significant role. In most cases, training involves an up-front investment, especially during the early phases of apprenticeships, which may later be recovered by firms (Wolter and Ryan 2011). If uncertainty about the immediate future is sufficiently high, firms may be more hesitant to incur such investment costs, even if productivity benefits from training exceed initial expenses over the course of the full contract duration. Furthermore, in Germany, an estimated 62% of training costs are wage costs of apprentices and only about 23% of training costs are associated with the wage costs of the instructors (Schönfeld et al. 2016), suggesting that opportunity cost reduction plays only a secondary role. To the extent that a recession is expected to be of short duration, however, firms may still hire new apprentices in 2020, if they expect that they will need to fill skilled-worker positions by 2023. Of course, even though our focus is on the demand side, with the firm’s decision to provide apprenticeships, it should not be left unmentioned that there is a supply side to this problem as well. Here, too, several effects are conceivable, as unemployment increases the supply of applicants for skilled positions or, on the other hand, interest in apprenticeship positions may decrease, as school graduates opt for further schooling instead of attempting to enter the VET labor market in times of increased unemployment (Weßling et al. 2015).

Based on these theoretical foundations, empirical studies mostly describe a positive (pro-cyclical) effect of business cycle developments on the number of apprenticeship contracts offered. This effect, however, is estimated to be small to moderate in size. Brunello (2009) provides a useful overview of empirical research. Among others, pro-cyclical behavior of apprenticeship markets has been shown for Norway (Askilden and Nilsen 2005), Denmark (Westergaard-Nielsen and Rasmussen 1999), or the United Kingdom (Merrilees 1983), predominantly using income, investment, order backlog, or unemployment statistics as independent variables. More recently, the Swiss cantonal context has attracted significant attention of researchers, again suggesting a pro-cyclical effect of economic developments on apprenticeships (Schweri and Müller 2008). The relationship is estimated to be small and largely overshadowed by more important demographic drivers (i.e. number of school graduates; Mühlemann et al. 2009). Lüthi and Wolter (2020a) provide the most recent contribution that analyzes Swiss data. While they conclude pro-cyclical effects as well, their evaluation provides a more nuanced picture. Emphasizing the importance of longer-term expectations, they argue that unemployment changes largely only lead to postponing of training activities, while GDP changes have a more sustained, moderate effect. Compared to Germany, however, the Swiss labor market may put greater emphasis on productivity-related training motivations (Mühlemann et al. 2010), leaving us with some question marks regarding the transferability of these findings to our empirical context.

When looking specifically at the German labor market, research has yet to produce conclusive interpretations of empirical data. In what is probably the study covering the longest time period to date, Baldi et al. (2014) investigate business cycle related drivers of apprenticeship contracts offered in Germany between 1999 and 2012. Their analysis shows that the effects of income growth and unemployment rates are small to none during what they refer to as “normal times” (p. 11). They do suggest, however, that this picture is a clearer one during more pronounced downturns, such as the 2008 financial crisis and its aftermath. Similar results are reported by Bellmann et al. (2014), who also investigate the impact of the 2008/2009 downturn on German apprenticeship numbers. Their findings indicate that while training activities declined during the crisis years, they did so irrespective of whether the firm was directly affected by the recession, driven more by the general macroeconomic outlook. They also argue that apprenticeships seem to be more robust to business cycle influences, based, however, on a short-term difference-in-differences estimation. Both contributions suggest that effects depend markedly on expectations about the general severity and duration of the economic downturn. Dietrich and Gerner (2007) offer the most directly applicable precedent to our study. In contrast to the previously mentioned publications, they do not look at actual economic developments, as measured by (lagged) income growth or unemployment rates, but instead look at the relationship between short-term business expectations and training provision. They show a pro-cyclical effect on the amount of training provided by firms, arguing mainly based on corresponding changes in assumptions about future transaction and opportunity cost.

We build on these prior contributions and offer the first long-term empirical analysis of business cycle effects on the German apprenticeship market that includes both the period of the Great Recession and initial data on economic expectations in the anticipated downturn caused by the Covid-19 pandemic. Lüthi and Wolter (2020b) have put forward a first projection of likely, decisively negative impacts of current economic developments on the Swiss apprenticeship market. They argue that especially bankruptcies may push the decrease of offered training positions beyond what we have observed during past downturns. Maier (2020) has developed a first scenario analysis for the German context, highlighting in particular the unevenness of the (again pro-cyclical) effect of ongoing developments. We offer an initial view of what can be expected for the German VET labor market based on current business cycle expectations.

## Method

In this section we first provide information about our data sources and descriptive statistics for our main variables of interest: demand for and supply of apprentices and the resulting number of new annual apprenticeship contracts in Germany, unfilled vacancies and unsuccessful applicants in the apprenticeship market, as well as two measures of company business cycle expectations. We further include descriptive statistics on the number of school leavers. Second, we discuss statistical models and our empirical estimation strategy.

### ifo Business Climate Index (BCI) and ifo Employment Barometer (EB)

**Fig. 1:**
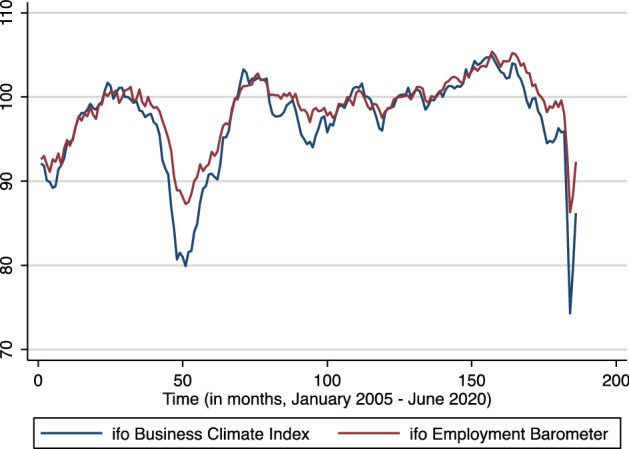
ifo Business Climate Index and ifo Employment Barometer (January 2005 to June 2020). Source: ifo Business Climate Index (*ifo Geschäftsklimaindex)*, normalized to the average of the year 2015, https://www.ifo.de/en/survey/ifo-business-climate-index, and ifo Employment Barometer *(ifo Beschäftigungsbarometer),* normalized to the average of the year 2015*,* https://www.ifo.de/en/survey/ifo-employment-barometer; see Sauer and Wohlrabe (2020) for detailed methodologies

Our main explanatory variable captures the expectation of firms about the business cycle at a particular point in time, as surveyed on a monthly basis by the ifo Institute. Figure 1 shows the development of our two indicators of interest, the Business Climate Index (BCI) and the Employment Barometer (EB) from January 2005 onwards. The BCI indicates companies’ assessment of the current business climate and their expectations for the next six months. It is one of Germany’s most relevant indicators about business cycle developments and a valid predictor of future GDP (Sauer and Wohlrabe 2020). The EB measures firms’ employment plans for the following three months and may therefore also be a good predictor of firm decisions to hire apprentices. As these measures of business cycle expectations are not available separately for all German states, we use the index at the national level. However, Sauer and Wohlrabe (2020, p.93) report that changes in the business expectation variables are highly correlated across states, where regional data is available.

Both indices reflect a significant drop during 2008 (the start of the financial crisis) and illustrate the subsequent recovery.^5^ In recent years, business cycle expectations continued to increase until August 2018, when both the BCI and the EB reached their peaks. They then decreased steadily but continuously, until the start of the coronavirus outbreak and the associated lockdown in Germany in March 2020. The BCI (EB) subsequently dropped to 74.3 (86.3) points in April 2020, their lowest values since January 2005. In May 2020, the indices recovered slightly to 79.5 (88.6) points and increased further in June 2020 to 86.2 (92.3). Thus, the average BCI (EB) is 80.1 (89.0) for the second quarter, which is 18.6 (11.7) points lower than the average of the second quarter in 2019.

As we only observe the yearly number of apprenticeship contracts, the indices need to be redefined on an annual basis as well. Table 1 illustrates the average annual change in BCI and EB, when considering the average of the first quarter (${\stackrel{-}{BCI}}_{Q1}$), the second quarter ${(\stackrel{-}{BCI}}_{Q2}$), or the first six months ${(\stackrel{-}{BCI}}_{Q1+Q2})$ of a particular year. As shown below, annual differences appear qualitatively rather similar. Our empirical estimates do not depend strongly on the exact definition of the relevant observation period to compute $\Delta \stackrel{-}{BCI}$ (and $\Delta \stackrel{-}{EB}$).^6^

**Table 1:** Yearly changes in the ifo Business Climate Index (BCI) and the ifo Employment Barometer (EB; 2005–2020)

Year	${\stackrel{-}{BCI}}_{Q1}$	${\stackrel{-}{BCI}}_{Q2}$	${\stackrel{-}{BCI}}_{Q1+Q2}$	$\Delta {\stackrel{-}{BCI}}_{Q1}$	$\Delta {\stackrel{-}{BCI}}_{Q2}$	$\Delta {\stackrel{-}{BCI}}_{Q1+Q2}$	${\stackrel{-}{EB}}_{Q2}$	$\Delta {\stackrel{-}{EB}}_{Q2}$
2005	91.3	89.5	90.4	n/a	n/a	n/a	92.0	n/a
2006	97.4	98.6	98.0	6.1	9.1	7.6	98.2	6.2
2007	100.5	100.7	100.6	3.1	2.1	2.6	100.0	1.8
2008	97.8	96.4	97.1	− 2.7	− 4.3	− 3.5	98.6	− 1.4
2009	80.8	82.4	81.6	− 17.0	− 14.0	− 15.5	88.7	− 9.9
2010	90.9	95.5	93.2	10.1	13.0	11.6	96.7	8.1
2011	101.9	102.1	102.0	11.0	6.7	8.8	102.3	5.6
2012	98.8	98.4	98.6	− 3.1	− 3.8	− 3.4	99.8	− 2.5
2013	97.2	96.4	96.8	− 1.7	− 2.0	− 1.8	97.8	− 1.9
2014	101.1	100.6	100.8	3.9	4.2	4.0	99.7	1.9
2015	98.8	100.1	99.5	− 2.3	− 0.4	− 1.4	100.0	0.2
2016	99.1	99.9	99.5	0.3	− 0.3	0.0	100.1	0.1
2017	101.4	103.0	102.2	2.3	3.1	2.7	102.8	2.7
2018	104.2	102.7	103.5	2.9	− 0.3	1.3	104.0	1.2
2019	99.4	98.7	99.1	− 4.9	− 4.0	− 4.4	100.6	− 3.4
2020	92.6	80.1	86.3	-6.8	− 18.6	− 12.7	89.0	− 11.7

Source: ifo Business Climate Index (*ifo Geschäftsklimaindex*, https://www.ifo.de/en/survey/ifo-business-climate-index) and ifo Employment Barometer (*ifo Beschäftigungsbarometer,* https://www.ifo.de/en/survey/ifo-employment-barometer)

### Demand and supply of apprentices

**Fig. 2:**
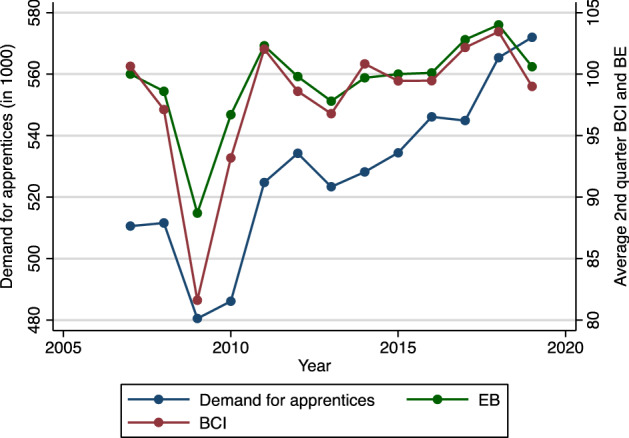
Demand for apprentices, ifo Business Climate Index (BCI), and ifo Employment Barometer (EB; 2007–2019). Source: ifo Business Climate Index *(ifo Geschäftsklimaindex*, https://www.ifo.de/en/survey/ifo-business-climate-index), and ifo Employment Barometer (*ifo Beschäftigungsbarometer,* https://www.ifo.de/en/survey/ifo-employment-barometer); Federal Employment Agency (statistics on the apprenticeship market)

A firm’s demand for apprentices is defined as the sum of the number of apprenticeship contracts and unfilled vacancies that firms post in a given year.^7^ Figure 2 shows that the demand for apprentices correlates strongly with the business climate in the period between 2007 and 2019.^8^ In particular, the decrease in demand for apprentices as a result of the financial crisis in 2009 and 2010 is clearly visible, as well as the subsequent increase during the economic recovery.

**Fig. 3:**
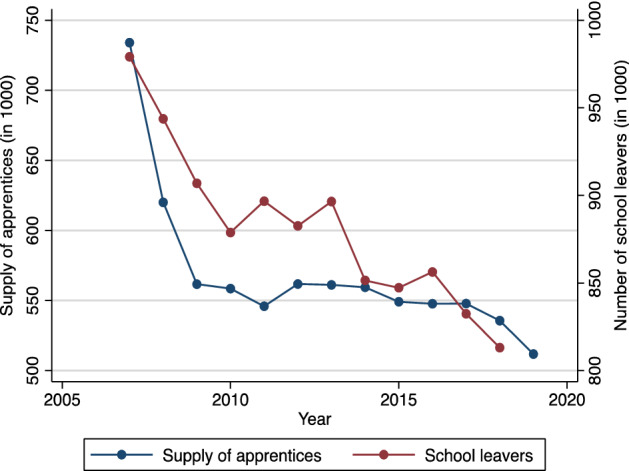
Supply of apprentices and number of school leavers (2007–2019). *Source:* Federal Employment Agency (statistics on the apprenticeship market), Federal Statistical Office (school leavers from general schools, excluding vocational schools)

The supply of apprentices is defined as the number of individuals that apply for an apprenticeship position in a particular year.^9^ Figure 3 shows that the supply correlates strongly with the number of school leavers. However, other factors such as the regional share of high school graduates and individual preferences may also play an important role.

### New apprenticeship contracts

In Germany, some firms already sign apprenticeship contracts early in the year, while others only find a suitable apprentice shortly before the start of training in August or September.^10^ Since many of the temporal aspects of the recruiting process are unclear or vary between firms and industries, it is not possible to precisely model in what month a firm’s business expectations are most relevant, when it comes to hiring apprentices. Furthermore, as we have described above, a firm can terminate apprenticeship contracts rather easily prior to the commencement of training and during its first one to four months. For that reason, we also estimate a regression to test whether changes in the Business Climate Index are associated with subsequent changes in the number of prematurely terminated apprenticeship contracts, however, without significant results (cf. Table 8).

**Fig. 4:**
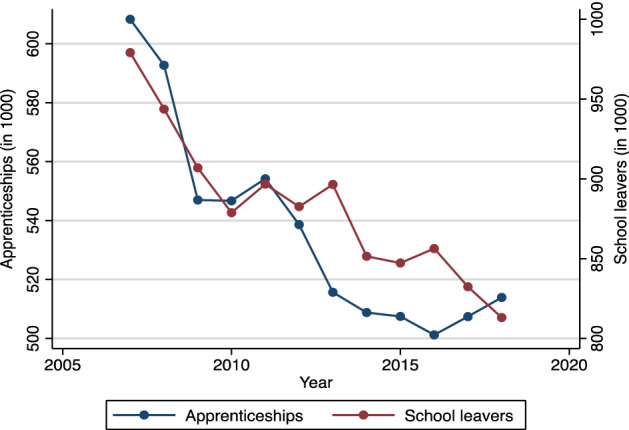
Number of firm-sponsored apprenticeship contracts and school leavers (2007–2018). *Sources:* Vocational Training Statistics of the statistical offices of the federal and state governments; Federal Statistical Office (school leavers from general schools, excluding vocational schools)

Figure 4 shows the development of the annual number of firm-sponsored apprenticeship contracts since 2007 and includes all such agreements as of 31 December 2018. Thus, any contract that was terminated after or even before the start of training would not be included in our data. The data further do not include publicly financed apprenticeship positions for people with disabilities, because their provision and governing regulations follow different principles compared to “regular” apprenticeship positions. Figure 4 illustrates that the number of firm-based apprenticeship contracts in Germany decreased from over 600,000 in 2007 to about 500,000 in 2016 and that this development correlates strongly with the annual number of school leavers. Moreover, we can observe an unusually strong decline in the number of apprenticeship contracts following the financial crisis from 2008 to 2009, when apprenticeships fell by 7.7% year over year. While demographic change (fewer school leavers) likely accounts for part of the decrease in the number of apprenticeship contracts throughout the observed period, the magnitude of the decline during the crisis years 2008 and 2009 suggests that economic conditions likely play a role as well. However, based on descriptive statistics, we cannot clearly distinguish the influence of demographic changes, business cycle fluctuations, other structural developments at the industry-level, or regional differences (such as the well-known matching problems that routinely leads to unfilled vacancies and unsuccessful applicants) by only looking at aggregate numbers for Germany.

For our empirical analysis we use the register of all apprenticeship contracts per year, starting in 2007. The contract information is collected by the regional chambers of commerce and then processed by the Federal Statistical Office. The final dataset is provided by the Federal Institute for Vocational Education and Training (BIBB).^11^ Reporting is mandatory for the regional chambers of industry and commerce, so that the register represents a full sample of all apprenticeships in Germany. Data include characteristics of the apprentices (contract holders) as well as regional and occupation-specific details. With this information, we construct a panel dataset that includes the number of new contracts in a given occupation, the state (*Bundesland*), and the contract year. A total of 321 occupations are recorded in the dual training system for all 16 federal states over a period of 12 years (from 2007 until 2018).^12^ The average number of apprenticeships per occupation by state is reported in the appendix (Tables 6 and 7). We also match the number of school leavers at the state-year level, in order to control for regional demographic change in our empirical analysis.

**Fig. 5:**
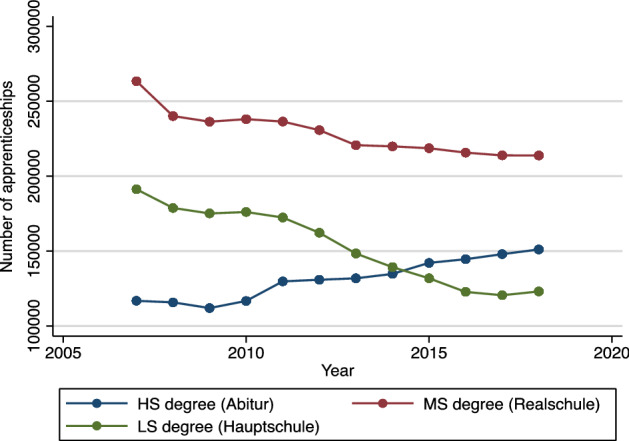
Number of apprenticeships by prior educational attainment (2007–2018). *Source:* Vocational Training Statistics of the statistical offices of the federal and state governments

A further notable characteristic of the German apprenticeship market is the difference in the development of apprenticeship contracts by applicants’ prior educational attainment, as presented in Fig. 5. A clear negative trend can be observed in the number of apprenticeship contracts for low-level school graduates (LS, *Hauptschule*) and graduates with a mid-level school qualification (MS, *Realschule*). Conversely, the number of apprenticeship contracts with individuals who have a high school degree (HS, *Abitur*), which also allows them to study at a German university, increased continuously from 2009 to 2018. With this in mind, we carry out our analysis at the state-level and further account for the occupational field at the 3-digit level. Differentiation at the occupational level is important, because there is substantial heterogeneity across apprenticeship occupations with regard to educational requirements, training duration, and future employment prospects (e.g., expected wage that an apprentice will earn as a skilled worker, as well as development opportunities). We further include distinct models, separated by educational subset of our sample (cf. Tables 9, 10, 11).

### Frictions in the German apprenticeship market: the dynamics of unfilled vacancies and unsuccessful applicants

So far, the descriptions provided are still missing one important dynamic: matching. The number of observed apprenticeship contracts in a particular year does not simply depend on aggregate demand and supply. Instead, it is crucial that the type of demand matches the type of supply in a given geographical or occupational area of the labor market. Resulting frictions, expressed in the number of unfilled vacancies and unsuccessful applicants, are important pieces of information that should be taken into account in the context of business cycle fluctuations.

**Fig. 6:**
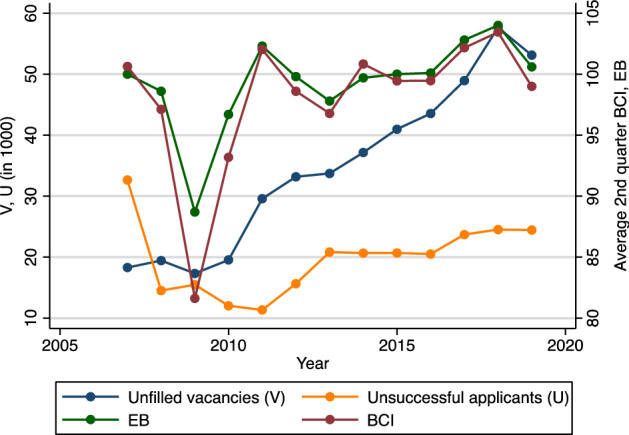
Number of unfilled vacancies, unsuccessful applicants, ifo Business Climate Index (BCI), and ifo Employment Barometer (EB; 2007–2019). Source: Apprenticeship market statistics 2007–2019, Federal Employment Agency (2020b), ifo Business Climate Index and ifo Employment Barometer

Figure 6 highlights that in the year following the financial crisis, both the number of unfilled apprenticeship vacancies as well as the number of unsuccessful applicants for apprenticeship positions were rather low, at less than 20,000 (unfilled vacancies) and 15,000 (unsuccessful applicants) respectively. In light of the economic recovery that started in 2010 and reached its peak in 2011, the number of unfilled vacancies began to increase, while the number of unsuccessful applicants initially dropped. As the economy remained strong through 2018, the number of unfilled vacancies rose substantially and came close to reaching 58,000 in 2018. At the same time, however, the number of unsuccessful applicants started to increase, despite the overall number of school leavers in Germany declining – an indication for an increased matching problem in the German apprenticeship market.

A more detailed analysis at the state-level reveals that the dynamics differ quite strongly across states. States in the South and East of Germany did not experience an increase in the share of unsuccessful applicants, while North Rhine-Westphalia, Hesse, Rhineland-Palatinate, as well as Hamburg and Schleswig–Holstein experienced increased matching problems, as both the share of unfilled vacancies and the share of unsuccessful applicants increased from 2010–2018 (Figs. 8, 9, 10, 11). Moreover, the level of unsuccessful applicants is lowest in the South of Germany (Baden-Wurttemberg and Bavaria).^13^

In addition to the described issues, there may also be many young applicants that fall outside of the definition of “unsuccessful”, as they were able to secure an alternative to an apprenticeship contract (e.g., a preparation year in order to close any skill gaps or other school-based alternatives). In 2019, on top of roughly 25,000 unsuccessful applicants, another 20,000 individuals were still actively searching for an apprenticeship, despite already having found an alternative to such a position (Federal Employment Agency 2020b).

### Statistical analysis

We follow Mühlemann et al. (2009), who estimate changes in the number of annual apprenticeships at the state-level. However, in our data, we are able to observe the number of apprenticeship positions not only for a particular state, but we also observe the occupational field within each state at the 5-digit level. Thus, our main dependent variable of interest is the logarithm of new apprenticeships in occupation $o$, state $s,$ and year $t$:

$${a}_{ost}={\upsilon }_{os} + {\mathbf{x}}_{st}^{^{\prime}}\beta +{\varepsilon }_{ost}$$

where ${\upsilon }_{so}$ accounts for unobserved heterogeneity at the state- and occupation-level, and $\mathbf{x}$ includes our main variables of interest ${(\stackrel{-}{BCI}}_{Q2t}{, \stackrel{-}{EB}}_{Q2t})$, as well as the logarithm of graduates ${g}_{st}$ from general schools in state $s$ in period $t$. The error term is represented by ${\varepsilon }_{ost}$.

We estimate first-differences regression models to account for unobserved factors at the state- and occupation-level. The differences in new annual (log) apprenticeship contracts are given by:

$${a}_{ost}-{a}_{ost, t-1}={\left({\mathbf{x}}_{st}-{\mathbf{x}}_{st,t-1}\right)}^{^{\prime}}\beta +({\varepsilon }_{ost}-{\varepsilon }_{ost-1})$$

To obtain consistent estimates, it is required that $E\left[\left({\varepsilon }_{ost}-{\varepsilon }_{ost-1}\right)|\left({\mathbf{x}}_{st}-{\mathbf{x}}_{st,t-1}\right)\right]=0$. This assumption is weaker compared to the strong exogeneity condition that is required to estimate an alternative fixed effects model, where $E\left[{\varepsilon }_{ost}|{\mathbf{x}}_{s1},\dots ,{\mathbf{x}}_{sT}\right]=0$. We estimate first-differences using OLS and report heteroskedasticity and autocorrelation-robust standard errors. We also include interaction terms of linear trends for 3-digit occupations and for East Germany, in order to account for occupation-specific trends and the stronger decrease in the number of apprenticeships in recent years in East German states (Tables 6 and 7).

Moreover, using the same framework, we estimate the demand for apprentices and the determinants of unfilled vacancies and unsuccessful applicants for the period of 2007 to 2019, using state-level data from the Federal Employment Agency (2020b). In particular, we estimate first differences in the log demand for apprentices, given by

$${d}_{st}-{d}_{st, t-1}={\left({\mathbf{x}}_{st}-{\mathbf{x}}_{st,t-1}\right)}^{^{\prime}}\beta +\left({\varepsilon }_{st}-{\varepsilon }_{st-1}\right),$$

where ${v}_{st}$ denotes the log demand for apprentices in state *s* at time *t*. Similarly, we estimate additional models using the log number of unfilled vacancies (${v}_{st}$) and unsuccessful applicants (${u}_{st}$) as dependent variables.

## Results and discussion

The following section reports the results of our first-differences regressions. Our main interest is to quantify the association between BCI or EB and the number of new apprenticeship contracts, conditional on controlling for the number of school graduates. Therefore, our key independent variables are $\Delta {\stackrel{-}{BCI}}_{Q2t,Q2t-1}$ and $\Delta {\stackrel{-}{EB}}_{Q2t,Q2t-1}$, which capture annual changes between second quarter average values of BCI and EB. We estimate the effect of these explanatory observations in subsequent models on log annual changes in the demand for apprentices, the number of unfilled vacancies and unsuccessful applicants, as well as in the number of apprenticeship contracts.

### Demand for apprentices and business cycle expectations

We first regress state-level demand for apprentices on BCI/EB and find, as expected, a positive and statistically significant association (see Table 2). According to Model 3, a 1-point increase in the BCI in period *t* (period *t-1*) is associated with a 0.37% (0.29%) increase in the demand for apprentices. To illustrate the economic significance, consider the demand for apprentices in 2009 compared to 2008: the BCI dropped by 14 points from 2008 to 2009, and by 4.3 points from 2007 to 2008.

**Table 2:** First-differences regression, demand for of apprentices (state-level, 2007–2019)

Log demand for apprentices $\Delta {d}_{st, t-1}$	Model 1	Model 2	Model 3	Model 4
$\Delta {\stackrel{-}{BCI}}_{Q2t,t-1}$	0.00318^a^	0.00322^a^	0.00371^a^	
	(0.000389)	(0.000418)	(0.000455)	
$\Delta {\stackrel{-}{BCI}}_{Q2t-1,t-2}$			0.00295^a^	
			(0.000633)	
$\Delta {\stackrel{-}{EB}}_{Q2t,t-1}$				0.00588^a^
				(0.000712)
$\Delta {\stackrel{-}{EB}}_{Q2t-1,t-2}$				0.00393^a^
				(0.000901)
$\Delta$ log number of school graduates ${g}_{st,t-1}$		0.135^a^	0.111^b^	0.115^b^
		(0.0422)	(0.0381)	(0.0382)
$\Delta$ log number of school graduates ${g}_{st-1,t-2}$			0.0963^b^	0.0949^b^
			(0.0388)	(0.0385)
Constant	0.00176	0.00440	0.00475	0.00293
	(0.00464)	(0.00386)	(0.00373)	(0.00382)
Observations	192	192	192	192
R-Squared	0.136	0.193	0.332	0.342

Heteroskedasticity and autocorrelation-robust standard errors in parentheses.  ^a^ Significant at the 1%-level; ^b^ significant at the 5%-level; ^c^ significant at the 10%-level. Data sources: Federal Employment Agency and BIBB-Survey of new apprenticeship contracts as of 30 September; ifo Business Climate Index and ifo Employment Barometer

Thus, according to our model, changes in business cycle expectations led to a $\frac{\partial \mathrm{d}}{\partial \mathrm{BIC}}=\left(-14\right)*0.00371 + \left(-4.3\right)*0.00295 = -\, 0.065 (\mathrm{or}-6.5\mathrm{\%})$ decrease in the demand for apprentices in 2009. Taking into account the most recent changes in BCI up to June 2020, our model predicts that firm demand for apprentices will decrease by $\frac{\partial \mathrm{d}}{\partial \mathrm{BIC}}=(-18.6)*0.00371 + (-4)*0.00295=-.081$, or $-8.1\mathrm{\%}$.^14^ Using the coefficients based on the ifo Employment Barometer, we find almost identical results, as $\frac{\partial \mathrm{d}}{\partial \mathrm{EB}}=(-11.7)*0.00588 + (-3.4)*0.00393=-.082$, or $-8.2\mathrm{\%}$.

### Unfilled vacancies, unsuccessful applicants, and business cycle expectations

In competitive markets, we expect that we do not observe unfilled vacancies or unsuccessful applicants, as prices (which are mainly apprentice wages in the context of apprenticeship training) would adjust and the market would eventually reach a new equilibrium after an unexpected macroeconomic shock (such as the financial crisis or the current coronavirus pandemic). However, as already illustrated in Fig. 6, there are considerable frictions in the German apprenticeship market that led to 53,000 unfilled vacancies in 2019 as well as almost 25,000 unsuccessful applicants.

Regressing unfilled vacancies on BCI at the state-level, we find a strong and positive effect. A 1-point increase in the BCI was associated with a 1.02% increase in unfilled vacancies. Moreover, when accounting for lagged effects, we find that a 1-point increase in the BCI in two consecutive years was associated with a 2.18% increase in unfilled vacancies (Models 1–3, Table 3). Conversely, we find no statistically significant association with changes in the BCI and changes in the number of unsuccessful applicants (Models 4–6, Table 3).^15^

**Table 3:** First-differences regression, unfilled vacancies and unsuccessful applicants (state-level, 2007–2019)

Dependent variable:	Log unfilled vacancies $\Delta {v}_{st, t-1}$			Log unsuccessful applicants $\Delta {u}_{st, t-1}$		
	Model 1	Model 2	Model 3	Model 4	Model 5	Model 6
$\Delta {\stackrel{-}{BCI}}_{Q2t,t-1}$	0.00876^b^	0.00868^b^	0.0102^a^	− 0.00217	− 0.00193	− 0.00206
	(0.00302)	(0.00308)	(0.00308)	(0.00562)	(0.00559)	(0.00587)
$\Delta {\stackrel{-}{BCI}}_{Q2t-1,t-2}$			0.0116^a^			0.000764
			(0.00338)			(0.00452)
$\Delta$ log number of school graduates ${g}_{st,t-1}$		− 0.247	− 0.338		0.699^b^	0.695^c^
		(0.277)	(0.248)		(0.304)	(0.344)
$\Delta$ log number of school graduates ${g}_{st-1,t-2}$			− 0.170			− 0.362
			(0.168)			(0.367)
Constant	0.0888^a^	0.0840^a^	0.0755^a^	− 0.0274^b^	− 0.0137	− 0.0206
	(0.0103)	(0.0106)	(0.0101)	(0.0105)	(0.0114)	(0.0119)
Observations	192	192	192	192	192	192
R-Squared	0.034	0.040	0.099	0.001	0.029	0.037

Heteroskedasticity and autocorrelation-robust standard errors in parentheses. ^a^ Significant at the 1%-level; ^b^ significant at the 5%-level;  ^c^ significant at the 10%-level. Data sources: Federal Employment Agency; ifo Business Climate Index and ifo Employment Barometer

### Apprenticeship contracts and business cycle expectations

Based on the previous results, we find that changes in BCI or EB are positively associated with firm demand for apprentices but are not associated with an increase in unsuccessful applicants for apprenticeship positions. We now turn to first estimating the association between BCI/EB and the number of apprenticeship contracts at the state-level, and subsequently at the occupation-state-level, in order to account for occupation-specific heterogeneity and developments.^16^

The results in Table 4 show a positive association between BCI/EB and the number of signed state-level apprenticeship contracts, although the coefficients for $\Delta {\stackrel{-}{BCI}}_{Q2t,t-1}$ and $\Delta {\stackrel{-}{EB}}_{Q2t,t-1}$ are somewhat lower than our estimates for labor demand (Table 2) and substantially lower compared to the regressions of unfilled vacancies (Table 3). Thus, our results suggest that business climate changes were not fully absorbed in the German apprenticeship market, instead resulting in an increased number of unfilled vacancies in recent years (when $\Delta {\stackrel{-}{BCI}}_{Q2t,t-1}>0)$. Our observation, that the number of unsuccessful applicants did not decrease substantially in recent years, can be explained (i) by matching problems and (ii) by the fact that many individuals, who are originally interested in apprenticeship training, do not end up being counted as unsuccessful applicants. Instead, labor market or schooling alternatives to apprenticeship training fill the void created by unsuccessful applications.^17^

**Table 4:** First-differences regression, new annual apprenticeship contracts (state-level, 2007–2019)

Dep.variable: log apprenticeship contracts $\Delta {a}_{st, t-1}$	Model 1	Model 2	Model 3	Model 4
$\Delta {\stackrel{-}{BCI}}_{Q2t,t-1}$	0.00281^a^	0.00285^a^	0.00327^a^	
	(0.000405)	(0.000451)	(0.000490)	
$\Delta {\stackrel{-}{BCI}}_{Q2t-1,t-2}$			0.00243^a^	
			(0.000701)	
$\Delta {\stackrel{-}{EB}}_{Q2t,t-1}$				0.00507^a^
				(0.000760)
$\Delta {\stackrel{-}{EB}}_{Q2t-1,t-2}$				0.00332^a^
				(0.00100)
$\Delta$ log number of school graduates ${g}_{st,t-1}$		0.139^a^	0.119^b^	0.122^b^
		(0.0466)	(0.0411)	(0.0415)
$\Delta$ log number of school graduates ${g}_{st-1,t-2}$			0.106^b^	0.104^b^
			(0.0390)	(0.0387)
Constant	− 0.00308	− 0.000355	0.000408	− 0.00119
	(0.00508)	(0.00415)	(0.00391)	(0.00402)
Observations	192	192	192	192
R-squared	0.095	0.149	0.248	0.253

Heteroskedasticity and autocorrelation-robust standard errors in parentheses. ^a^ Significant at the 1%-level; ^b^ significant at the 5%-level; ^c^ significant at the 10%-level. Data sources: Vocational training statistics of the statistical offices of the federal and state governments, ifo Business Climate Index and ifo Employment Barometer

However, the number of observations at the state-level is relatively small and we are not able to account for occupation-specific heterogeneity by using only aggregate statistics. For that reason, we make use of more fine-grained data at the occupation-state-level (vocational training statistics of the statistical offices of the federal and state governments). Regression results are presented in Table 5.

**Table 5:** First-differences regression (occupation-state-level, 2007–2018)

Dependent variable: log number of apprenticeship contracts $\Delta {a}_{ost, t-1}$	Model 1	Model 2	Model 3	Model 4	Model 5	Model 6
$\Delta {\stackrel{-}{BCI}}_{Q2t,t-1}$	0.00211^a^	0.00215^a^	0.00284^a^	0.00280^a^	0.00279^a^	
	(0.000429)	(0.000457)	(0.000457)	(0.000459)	(0.000459)	
$\Delta {\stackrel{-}{BCI}}_{Q2t-1,t-2}$			0.00201^a^	0.00186^a^	0.00187^a^	
			(0.000385)	(0.000388)	(0.000388)	
$\Delta {\stackrel{-}{EB}}_{Q2t,t-1}$						0.00457^a^
						(0.000650)
$\Delta {\stackrel{-}{EB}}_{Q2t-1,t-2}$						0.00245^a^
						(0.000576)
$\Delta$ log number of school graduates ${g}_{st,t-1}$		0.215^a^	0.211^a^	0.200^a^	0.191^a^	0.194^a^
		(0.0230)	(0.0251)	(0.0252)	(0.0251)	(0.0251)
$\Delta$ log number of school graduates ${g}_{st-1,t-2}$			0.0908^a^	0.0742^a^	0.0528^a^	0.0568^a^
			(0.0246)	(0.0247)	(0.0250)	(0.0250)
Trend*East Germany					− 0.00233^a^	− 0.00273^a^
					(0.000411)	(0.000272)
Occupation-level trends	No	No	No	Yes	Yes	Yes
Constant	− 0.0250^a^	− 0.0208^a^	− 0.0244^a^	− 0.118^a^	− 0.114^b^	− 0.114^b^
	(0.00145)	(0.00147)	(0.00159)	(0.0450)	(0.0450)	(0.0477)
Observations	38,880	38,880	34,160	34,160	34,160	34,160
R-squared	0.001	0.003	0.005	0.028	0.028	0.029

Heteroskedasticity and autocorrelation-robust standard errors in parentheses. ^a^ Significant at the 1%-level; ^b^ significant at the 5%-level; ^c^ significant at the 10%-level. Data sources: Vocational training statistics of the statistical offices of the federal and state governments, ifo Business Climate Index (BCI), ifo Employment Barometer (EB)

In order to estimate the effect of the financial crisis, which started to affect BCI and EB in the fall of 2008, we estimate $\Delta {a}_{os\mathrm{2009,2008}}={\beta }_{1}*\Delta {\stackrel{-}{BCI}}_{Q2 2009,2008}+{\beta }_{2}*\Delta {\stackrel{-}{BCI}}_{Q2 2008,2007}$, which is the change in the number of apprenticeship contracts from 2008 to 2009 due to changes in the BCI (applied correspondingly for EB). According to our analysis (Model 5, Table 5), the drop in the Business Climate Index from 2008 to 2009 (which was -14 index points, cf. Table 1) was therefore associated with a $0.00279*\left(-14.0\right)=-3.9\%$ decrease in the number of apprenticeship positions. However, as the index already dropped from the second quarter in 2007 to the second quarter 2008 by 4.3 index points, this caused an additional decrease in apprenticeship contracts of $0.00187*\left(-4.3\right) = - 0.8 \%$. In sum, changes in the BCI between 2007 and 2009 are therefore associated with a 4.7% decrease in the number of apprenticeship contracts (ceteris paribus). Our estimation using EB as the key indicator for expected business cycle changes yields larger coefficients, but as the Employment Barometer seems somewhat less sensitive to economic developments (leading to a smaller decline, relative to the BCI), total results are very similar (4.9% decrease).^18^ As the overall decrease in the number of new apprenticeships from 2008 to 2009 was 7.7% (cf. BIBB 2019), changes in the BCI can explain 61% of that decrease in the number of apprenticeship contracts. Clearly, demographic change was a second relevant influencing factor that can, in part, explain the decrease in apprenticeships in Germany since 2008.

Our analysis further shows that apprentices with a high school degree (and, to a lesser extent, individuals with a low-level school degree, i.e., *Hauptschule*) are more strongly affected by changes in the business cycle (cf. Tables 9, 10, 11; offering additional evidence for suggestions by Maier 2020). By contrast, apprenticeship contracts with individuals that have obtained mid-level school degrees (*Realschule*) seem to remain largely unaffected by changes in the business climate from 2007–2018. We propose that this is primarily driven by principal differences in the availability of labor market or schooling alternatives (to apprenticeships) that are open to the respective educational segment. As prospects for available apprenticeship positions worsen in an economic downturn, high school graduates may increasingly opt for alternative career paths, such as university programs. By contrast, low-level school graduates can likely not evade negative effects of crises as easily and, instead, suffer from them directly. Two primary mechanisms are conceivable. Firstly, as competition for available apprenticeships increases, other applicants with higher schooling degrees may increasingly fill positions with usually less demanding schooling requirements. Secondly, firms that, during “normal” times, represent the primary employers for applicants with low-level schooling qualifications may be exposed to higher amounts of economic risks and financial strain during times of crisis, causing them to reduce their apprenticeship offering disproportionately, or drop out of the market altogether. Mid-level school graduates, on the other hand, seem to be able to potentially benefit from these movements in the other two educational segments, leaving them less exposed to business cycle developments.

### What is the predicted effect of Covid-19 on the German apprenticeship market?

**Fig. 7:**
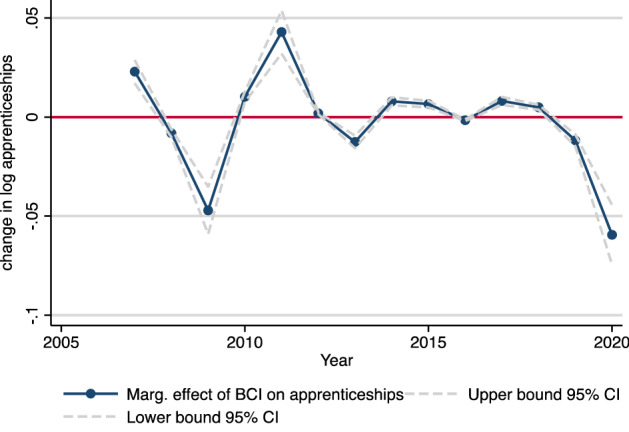
Marginal effects of the BCI (ceteris paribus) on the number of new apprenticeships (2007–2018) and predicted effects for the years 2019 and 2020. Notes: Blue line indicates to what extent changes in the ifo BCI (average value of the second quarter of each year) affect the annual number of new apprenticeship contracts signed by the end of the same year. Out-of-sample forecasts for the years 2019 and 2020 that include current ifo BCI data for January 2019 – June 2020. Data sources: Vocational training statistics of the statistical offices of the federal and state governments, ifo Business Climate Index

**Fig. 8:**
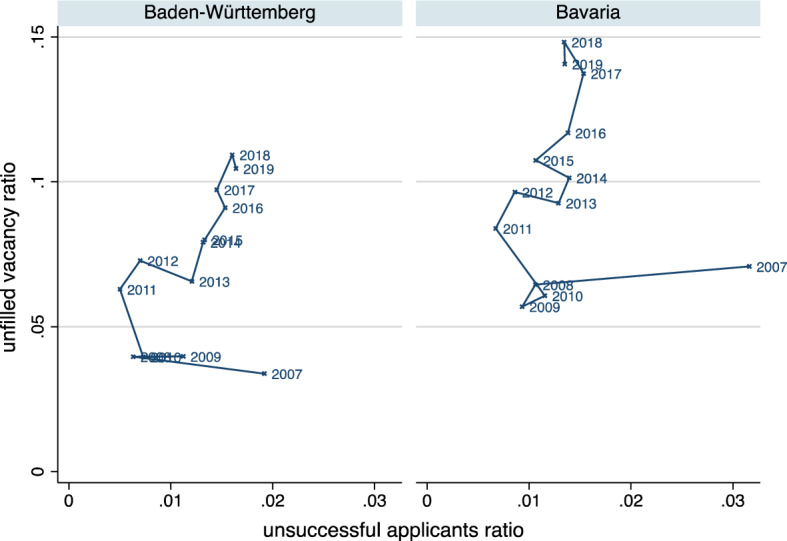
Beveridge curves for South German states (2007–2019). Data sources: Vocational training statistics of the statistical offices of the federal and state governments

**Fig. 9:**
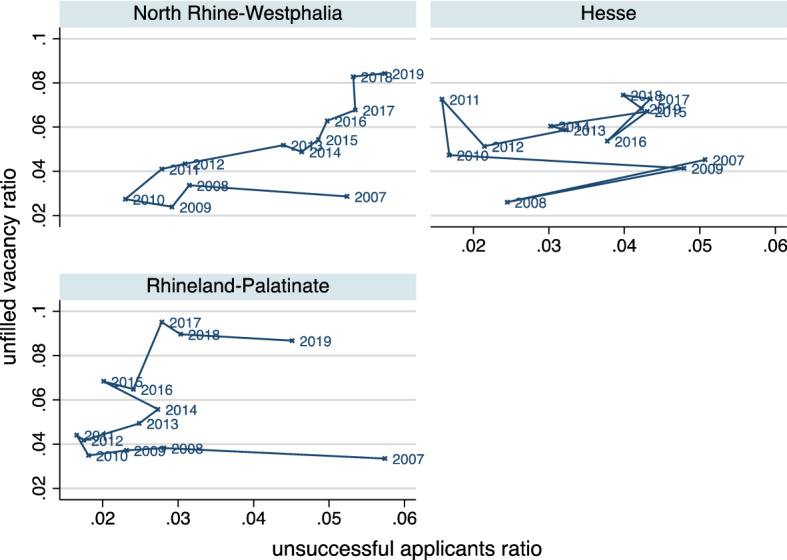
Beveridge curves for West German states (2007–2019). Data sources: Vocational training statistics of the statistical offices of the federal and state governments

**Fig. 10:**
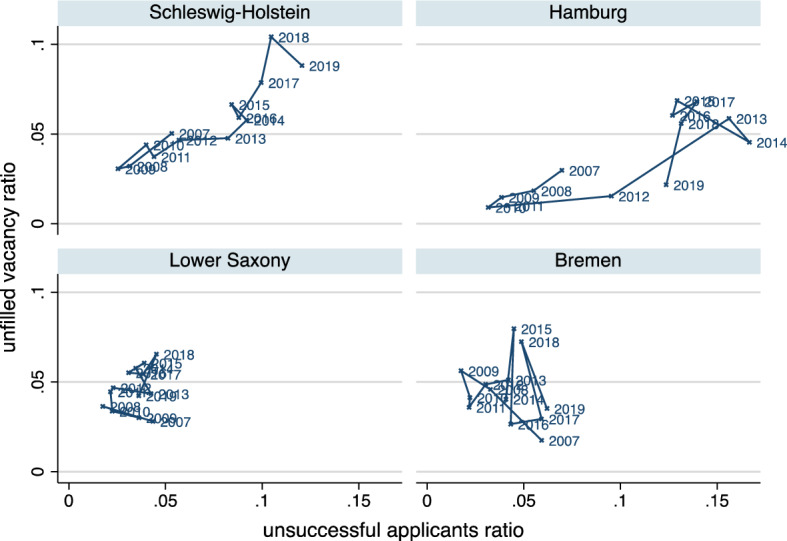
Beveridge curves for North-Western German states (2007–2019). Data sources: Vocational training statistics of the statistical offices of the federal and state governments

**Fig. 11:**
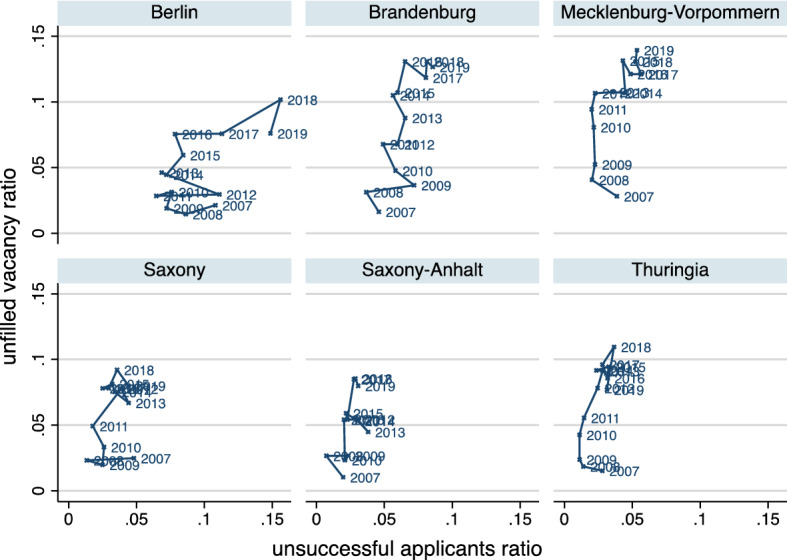
Beveridge curves for East German states (2007–2019). Data sources: Vocational training statistics of the statistical offices of the federal and state governments

Figure 7 plots the change in the log number of apprenticeship contracts due to changes in the BCI.^19^ To the extent that we can extrapolate our findings from the 2007–2018 period (i.e., our estimates for coefficients of $\Delta {\stackrel{-}{BCI}}_{Q2t,t-1}$ and $\Delta {\stackrel{-}{BCI}}_{Q2t-1,t-2}$ in Table 5, Model 5), we would expect a decline in the number of apprenticeship contracts in 2020 of $\Delta {a}_{os\mathrm{2020,2019}}={\beta }_{1}*\Delta {\stackrel{-}{BCI}}_{Q2 2020,2019}+{\beta }_{2}*\Delta {\stackrel{-}{BCI}}_{Q2 2019,2018}= 0.00279*\left(-18.6\right)+0.00187*\left(-4\right)\cong - 0.059$, or − 5.9% (95% CI: ± 0.015; ceteris paribus compared to 2019). This corresponds, on average, to 30,300 (95% CI: ± 7800) fewer apprenticeship training positions compared to 2019, largely due to the recent Covid-19-induced change in expectations about the business climate.^20^ The corresponding estimate based on the coefficients of the ifo Employment Barometer (EB) in Model 6 (Table 5) is slightly higher, as $(-3.4)*0.00245 +(-13.3)*0.00457= - 0.062$, or − 6.2%.

While our results cannot be directly compared to recent estimations put forth by Maier (2020), they seem to solidify the expected direction. Maier (2020), however, does not base his predictions on current data on business cycle expectations, but rather on estimates about the development of German GDP in 2020. Such projections range from anywhere between − 2.8% and − 11.2%, and, therefore, different scenarios for the development of apprenticeship contracts are estimated (Maier 2020, p. 7). Maier (2020) reports that in a scenario of a 7% decrease in GDP, the estimated number of apprenticeship contracts decreases by about 30,000 contracts to a total of 480,600 apprenticeships (− 5.8%), which corresponds very closely to our results, while a 11.2% (2.8%) decrease in the GDP would be associated with a 9.3% (2.3%) decrease in apprenticeship positions.

## Conclusions

Empirical evidence for Germany shows that the apprenticeship market is susceptible to business cycle fluctuations. Thus, the large and unexpected economic shock associated with the Covid-19 pandemic and the lockdown measures that came in its wake are likely to affect the German apprenticeship market in 2020. Based on our analysis of the association between two indicators of business cycle expectations (the ifo Business Climate Index and the ifo Employment Barometer) and subsequent apprenticeship contracts from 2007 to 2018, which also includes the financial crisis in 2008/2009, we predict that current company expectations about business cycle developments will lead to a reduction in the number of apprenticeship contracts in August/September 2020 by 6% or 30,000 positions (95% CI: ± 8000). Moreover, our results suggest that the effect of the crisis will be more pronounced for the demand for apprentices (i.e., the sum of apprenticeship contracts and unfilled vacancies), where our regression model predicts a − 9.1% decrease based on German firms’ current business cycle expectations (as measured through the BCI). This is driven by frictions in the apprenticeship market. In 2019, total demand for apprentices exceeded total supply and resulted in 53,000 unfilled vacancies. Therefore, the effect of Covid-19 on the number of apprenticeship contracts will likely be less severe than if we had had a situation of excess supply prior to the start of the current crisis, as was the case in 2008 (before the start of the financial crisis). In a number of occupations and regions in 2020, many firms would simply not have been able to successfully fill their vacancies in the absence of the coronavirus crisis, in part because demographic changes continue to put downward pressure on the number of school leavers. Furthermore, we did not find any statistically significant association between changes in the BCI/EB and changes in the number of unsuccessful applicants in the period between 2007 and 2019. Thus, there is some hope that the number of unsuccessful applicants in the apprenticeship market will not increase as drastically in occupations, where demand exceeded supply in 2019. Nonetheless, there will likely be a strong increase in the number of individuals that have to accept alternative educational arrangements, such as preparatory courses to increase the chances to secure an apprenticeship position in 2021 (and thus compete for apprenticeship positions with next year’s school leavers). Our analysis shows that this will apply especially to school graduates with lower school attainment (*Hauptschule*), because those with a high school degree (*Abitur*) will have other options available to them, for example, to pursue a tertiary degree.

A cautionary note appears appropriate for all our forward-looking results. While our econometric model offers predictions based on historic market information, it cannot provide definitive answers to questions about how the crisis and associated consequences for apprenticeships will unfold. We do, however, provide initial means to estimate and thus prepare for the likely impact of Covid-19 on the German apprenticeship market. Furthermore, the benefit of our approach is that the analysis can easily be updated, once more data become available. Our predictions remain a “best guess” estimate, based on the information available at the time.

Finally, the current pandemic and the resulting economic downturn coincide with significant regulatory changes that have introduced a minimum wage for German apprentices at the beginning of 2020. Initial studies on the likely impact of the specific level of the German minimum wage for apprentices suggest that it is largely small companies and those in the eastern German states that would see rising costs (Wenzelmann and Pfeifer 2018). These influences may be felt in addition to the expected effects we have described and while we do not discuss them in greater detail, they should provide ample material for future research efforts.

## Figures and Tables

**Fig. 12:**
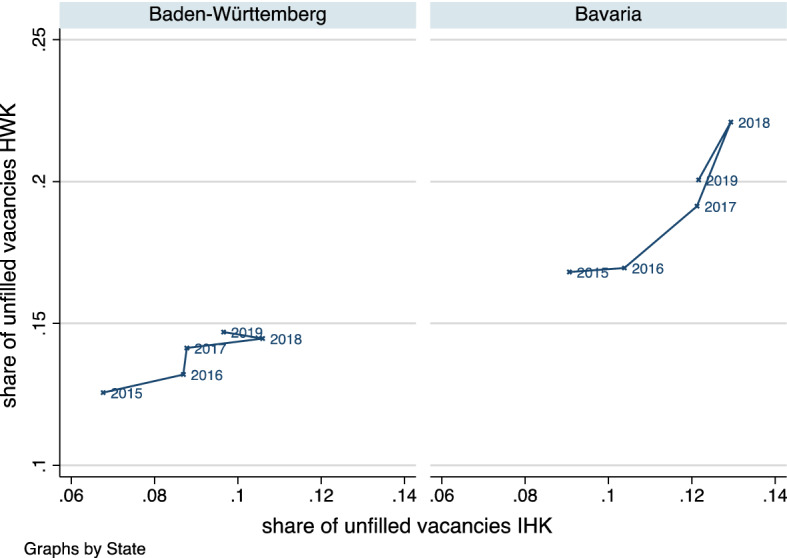
Share of unfilled vacancies in craft (HWK) and industry and trade (IHK) for South German states, 2015–2019. Data sources: Vocational training statistics of the statistical offices of the federal and state governments

**Fig. 13:**
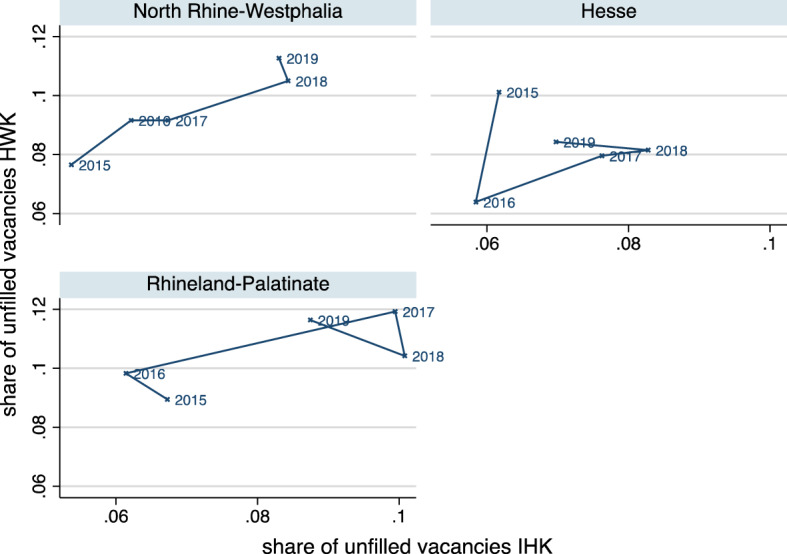
Share of unfilled vacancies in craft (HWK) and industry and trade (IHK) for West German states, 2015–2019. Data sources: Vocational training statistics of the statistical offices of the federal and state governments

**Fig. 14:**
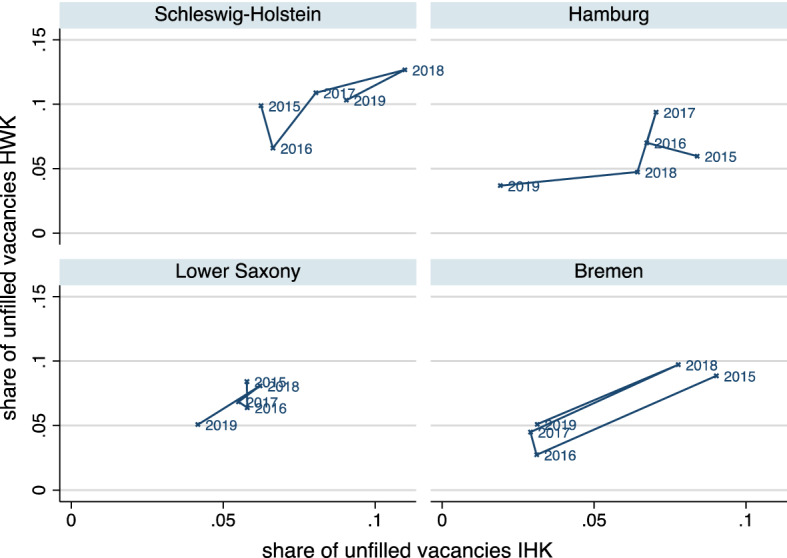
Share of unfilled vacancies in craft (HWK) and industry and trade (IHK) for North-Western German states, 2015–2019. Data sources: Vocational training statistics of the statistical offices of the federal and state governments

**Fig. 15:**
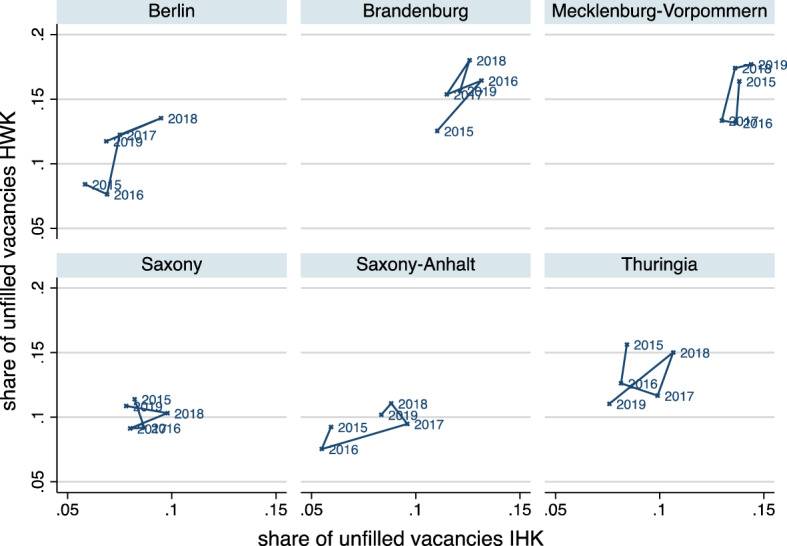
Share of unfilled vacancies in craft (HWK) and industry and trade (IHK) for East German states, 2015–2019. Data sources: Vocational training statistics of the statistical offices of the federal and state governments

## Data Availability

The data at the state-level is publicly available on the website of the Federal Employment Agency (https://statistik.arbeitsagentur.de/Navigation/Statistik/Statistik-nach-Themen/Ausbildungsstellenmarkt/zu-den-Daten/zu-den-Daten-Nav.html). The data at the occupational-level is provided by the BIBB and not publicly available data due to data protection laws. The ifo business cycle indicators are publicly available, as indicated in the text.

## Appendix

See Tables 6, 7, 8, 9, 10 and 11.

**Table 6:** Number of new apprenticeships by occupation (2007–2012)

	Number of apprenticeships by occupation											
	2007		2008		2009		2010		2011		2012	
State	Mean	SD	Mean	SD	Mean	SD	Mean	SD	Mean	SD	Mean	SD
Schleswig–Holstein	94	193	92	189	90	181	86	182	87	180	86	178
Hamburg	68	134	68	135	63	124	67	134	63	131	63	127
Lower Saxony	226	481	219	476	209	453	215	472	227	499	216	470
Bremen	32	58	32	58	31	54	32	55	31	56	32	58
North Rhine-Westphalia	458	1075	436	991	412	957	426	1003	429	1017	420	996
Hesse	162	383	155	362	147	348	144	344	147	344	148	340
Rhineland-Palatinate	125	277	117	254	113	249	114	249	111	247	110	239
Baden-Württemberg	284	641	280	639	269	610	264	606	277	634	265	622
Bavaria	359	836	356	841	322	760	326	779	332	795	333	774
Saarland	47	90	45	85	45	85	43	80	44	78	43	80
Berlin	100	231	89	207	86	194	83	187	79	175	81	178
Brandenburg	77	166	69	145	60	127	56	117	49	101	46	94
Mecklenburg-Vorpommern	76	164	65	132	51	101	47	90	42	78	41	75
Saxony	115	239	98	207	89	193	79	171	74	158	69	136
Saxony-Anhalt	81	164	74	148	62	125	57	115	52	106	51	98
Thuringia	73	142	66	129	56	108	51	100	46	88	46	85

Data sources: Vocational training statistics of the statistical offices of the federal and state governments

**Table 7:** Number of new apprenticeships by occupation (2013–2018)

	Number of apprenticeships by occupation											
	2013		2014		2015		2016		2017		2018	
State	Mean	SD	Mean	SD	Mean	SD	Mean	SD	Mean	SD	Mean	SD
Schleswig–Holstein	81	170	83	172	82	169	87	174	84	170	84	165
Hamburg	61	124	61	123	62	126	62	123	61	120	62	120
Lower Saxony	205	453	211	460	204	445	204	440	211	445	206	439
Bremen	31	55	31	55	31	55	31	55	31	53	30	54
North Rhine-Westphalia	415	981	402	948	401	943	400	927	408	933	408	928
Hesse	140	317	140	312	138	308	135	301	141	303	141	308
Rhineland-Palatinate	104	225	105	228	103	217	104	218	100	214	102	212
Baden-Württemberg	259	598	258	592	264	597	262	588	267	591	271	593
Bavaria	321	755	319	745	321	745	316	735	326	748	331	759
Saarland	42	74	40	70	41	71	39	69	39	68	37	64
Berlin	74	161	72	152	71	148	70	146	70	142	72	143
Brandenburg	44	86	43	83	44	86	43	83	42	84	44	86
Mecklenburg-Vorpommern	38	72	39	71	38	71	39	69	39	68	40	71
Saxony	68	134	69	136	69	134	71	135	75	141	75	143
Saxony-Anhalt	47	93	48	94	48	92	48	93	45	87	47	90
Thuringia	44	82	43	80	45	80	44	78	45	81	46	83

Data sources: Vocational training statistics of the statistical offices of the federal and state governments

**Table 8:** First-differences regression, prematurely terminated training contracts (state-level)

Dependent variable (log):$\Delta {tc}_{st, t-1}$	Model 1	Model 2
$\Delta {\stackrel{-}{BCI}}_{Q2t,t-1}$	− 0.0000517	0.0000429
	(0.000381)	(0.000373)
$\Delta$ log number of school graduates ${g}_{st,t-1}$		0.00125
		(0.0445)
Constant	0.000373	− 0.00333
	(0.00535)	(0.00599)
Observations	176	174
R-squared	0.148	0.428

Heteroskedasticity and autocorrelation-robust standard errors in parentheses. ^a^ Significant at the 1%-level; ^b^ significant at the 5%-level; ^c^ significant at the 10%-level. Data sources: Vocational training statistics of the statistical offices of the federal and state governments, ifo Business Climate Index

**Table 9:** First-differences regression (occupation-state-level, apprentices with *a high school degree*)

Dependent variable (log):$\Delta {a}_{ost, t-1}$	Model 1	Model 2	Model 3	Model 4	Model 5
$\Delta {\stackrel{-}{BCI}}_{Q2t,t-1}$	0.00196^a^	0.00247^a^	0.00273^a^	0.00267^a^	0.00257^b^
	(0.000592)	(0.000590)	(0.000640)	(0.000644)	(0.000645)
$\Delta$ log number of school graduates ${g}_{st,t-1}$		0.179^a^	0.178^a^	0.177^a^	0.160^a^
		(0.0182)	(0.0184)	(0.0184)	(0.0186)
$\Delta {\stackrel{-}{BCI}}_{Q2t-1,t-2}$			0.00181^a^	0.00165^a^	0.00163^a^
			(0.000552)	(0.000558)	(0.000557)
$\Delta$ log number of school graduates ${g}_{st-1,t-2}$			0.0634^a^	0.0617^a^	0.0433^a^
			(0.0174)	(0.0174)	(0.0178)
Trend*East Germany					− 0.0527^b^
					(0.00426)
Occupation-level trends	No	No	No	Yes	Yes
Constant	0.0301^a^	0.0299^a^	0.0287^a^	− 0.0488	− 0.0366
	(0.00197)	(0.00195)	(0.00215)	(0.0343)	(0.0391)
Observations	27,367	27,367	24,669	24,669	24,669
R-squared	0.001	0.005	0.005	0.021	0.023

Heteroskedasticity and autocorrelation-robust standard errors in parentheses. ^a^ Significant at the 1%-level; ^b^ significant at the 5%-level; ^c^ significant at the 10%-level. Data sources: Vocational training statistics of the statistical offices of the federal and state governments, ifo Business Climate Index

**Table 10:** First-differences regression (occupation-state-level, apprentices with *a mid-level school* degree)

Dependent variable (log):$\Delta {a}_{ost, t-1}$	Model 1	Model 2	Model 3	Model 4	Model 5
$\Delta {\stackrel{-}{BCI}}_{Q2t,t-1}$	0.000842	0.000830	0.0000311	− 0.00000704	0.0000177
	(0.000526)	(0.000526)	(0.000565)	(0.000568)	(0.000569)
$\Delta \mathrm{log number}$ of school graduates ${g}_{st,t-1}$		0.0855^a^	0.0561^b^	0.0517^b^	0.0498^b^
		(0.0235)	(0.0253)	(0.0254)	(0.0254)
$\Delta {\stackrel{-}{BCI}}_{Q2t-1,t-2}$			− 0.000123	− 0.000285	− 0.000281
			(0.000477)	(0.000482)	(0.000482)
$\Delta$ log number of school graduates ${g}_{st-1,t-2}$			0.158^a^	0.149^a^	0.129^b^
			(0.0220)	(0.0222)	(0.0225)
Trend*East Germany					− 0.0252^a^
					(0.00327)
Occupation-level trends	No	No	No	Yes	Yes
Constant	− 0.0288^a^	− 0.0271^a^	− 0.0187^a^	− 0.297^a^	− 0.297^a^
	(0.00164)	(0.00168)	(0.00180)	(0.0467)	(0.0467)
Observations	33,754	33,754	30,129	30,129	30,129
R-squared	0.000	0.001	0.001	0.014	0.015

Heteroskedasticity and autocorrelation-robust standard errors in parentheses. ^a^ Significant at the 1%-level; ^b^ significant at the 5%-level; ^c^ significant at the 10%-level. Data sources: Vocational training statistics of the statistical offices of the federal and state governments, ifo Business Climate Index

**Table 11:** First-differences regression (occupation-state-level, apprentices with *low-level school* degree)

Dependent variable (log):$\Delta {a}_{ost, t-1}$	Model 1	Model 2	Model 3	Model 4	Model 5
$\Delta {\stackrel{-}{BCI}}_{Q2t,t-1}$	0.00149^b^	0.00126^b^	0.00142^b^	0.00143^b^	0.00144^b^
	(0.000621)	(0.000627)	(0.000656)	(0.000663)	(0.000663)
$\Delta$ log number of school graduates ${g}_{st,t-1}$		0.109^a^	0.0657^c^	0.0455	0.0406
		(0.0372)	(0.0398)	(0.0400)	(0.0403)
$\Delta {\stackrel{-}{BCI}}_{Q2t-1,t-2}$			0.000535	0.000449	0.000447
			(0.000580)	(0.000585)	(0.000585)
$\Delta$ log number of school graduates ${g}_{st-1,t-2}$			0.0342	0.0142	0.0160
			(0.0383)	(0.0386)	(0.0386)
Trend*East Germany					0.0178^b^
					(0.00472)
Occupation-level trends	No	No	No	Yes	Yes
Constant	− 0.0348^a^	− 0.0292^a^	− 0.0297^a^	0.554	0.554
	(0.00196)	(0.00264)	(0.00315)	(0.388)	(0.388)
Observations	26,851	26,851	24,046	24,046	24,046
R-squared	0.000	0.001	0.000	0.013	0.014

Heteroskedasticity and autocorrelation-robust standard errors in parentheses. ^a^ Significant at the 1%-level; ^b^ significant at the 5%-level; ^c^ significant at the 10%-level. Data sources: Vocational training statistics of the statistical offices of the federal and state governments, ifo Business Climate Index

See Figs. 8, 9, 10, 11, 12, 13, 14, 15

## Footnotes

1 US unemployment numbers include a misclassification error reported by USBLS (2020a). This impacts data for March, April, May, and June. The degree of misclassification is expected to have decreased significantly in June. However, numbers likely remain understated.

2 Hippach-Schneider and Huismann (2016) provide a useful and more comprehensive overview.

3 Research has described the minimum wage as an “obvious barrier to job training” (Leighton and Mincer 1981, p. 158) early on and argued that employers may, as a result, seek to increase employees’ time allocated to productive activities (Hashimoto 1982), which can be problematic during a recession. While this development is not the focus of our undertaking, the cost increases it may potentially represent, could conceivably add to the current dynamic in some cases.

4 Moreover, the Federal Employment Agency (*Bundesagentur für Arbeit*) publishes monthly statistics on the number of advertised vacancies, registered applicants, and successfully signed contracts in the German apprenticeship market (https://statistik.arbeitsagentur.de/Navigation/Statistik/Statistik-nach-Themen/Ausbildungsstellenmarkt/Ausbildungsstellenmarkt-Nav.html) for the months of January to September.

5 However, firms’ employment plans, as measured in the EB, tend to be less volatile compared to the assessment of the business climate. This can be explained by frictions in the German labor market (employment protection legislation) as well as the possibility for firms to apply for short-time work compensation (*Kurzarbeitergeld*), both of which typically prevent firms from immediate layoffs during an economic crisis.

6 For reasons of brevity, we refrain from always reporting all of our results of both BCI and EB, but we include a model that uses the EB as an independent variable in our main regression tables (Tables 2, 3, 4, and 5). As it turns out, our results are very similar and robust irrespective of which of the two indicators we use in our regression models.

7 Note that unfilled vacancies only include registered vacancies with the Federal Employment Agency, and not the entire population of unfilled vacancies in Germany. Although no statistics exist that include the population of all open positions in Germany, registered vacancies with the Federal Employment Agency likely correlate strongly with the total number of vacancies in Germany over time.

8 The state-level data is published by the Federal Employment Agency and refers to 30 September of each year. Note that contrary to this, all data at the occupation-level (Tables 5, 6, 7, 9, 10, 11) refer to 31 December of each year and, thus, also cover all contracts that were signed or terminated between 30 September and the end of the respective year.

9 Note that the supply is defined as the sum of the number of apprenticeship contracts and the number of unsuccessful applicants who are registered with the Federal Employment Agency, and not the entire population of unsuccessful applicants in Germany.

10 The exact start of an apprenticeship program varies by state. By June 2020, a total of 479,200 apprenticeship positions were advertised by training firms (− 8.9% compared to 2019) and 241,000 individuals had successfully signed an apprenticeship contract (− 15.9% compared to 2019), cf. Federal Employment Agency (2020b).

11 For more details on the data contents, see Destatis (2012). For a discussion of the research potential of this data, refer to Rohrbach-Schmidt and Uhly (2016).

12 Microdata for 2019 were not yet available at the time of publication.

13 Within states, there are also some differences in the development of unfilled vacancies that depend on occupational fields (crafts sector vs. industry and trade), as indicated in Figures A5-A8.

14 Our prediction is close to currently observed changes in the demand for apprentices compared to the previous year, which decreased by 8.9% (apprenticeship market statistics, June 2020; Federal Employment Agency 2020b).

15 We also run the same regressions using EB rather than BCI as independent variables. The results are very similar and not included for reasons of brevity. The results are available from the authors upon request.

16 Data at the occupation-level within states are only available until the end of 2018.

17 We also ran similar regressions of the number of unsuccessful applicants that had already found an alternative (as published by the Federal Employment Agency) on the BCI for the period of 2007–2019, but found no evidence for a statistically significant association.

18 Changes in the Employment Barometer (EB) amount to − 1.4 from 2007 to 2008 and − 9.9 from 2008 to 2009 (Table 1). Thus, based on the coefficients in Model 6 (Table 5), changes in the EB suggest a 4.86% decrease in the number of apprenticeship contracts from 2008 to 2009.

19 The state- and occupation-level number of apprenticeship contracts are not yet available for 2019, even though an estimate of the total number of apprenticeship positions in 2019 (510,710 firm-sponsored apprenticeships) has already been published (Berufsbildungsbericht 2019; BIBB 2019).

20 A further effect of changes in business cycle expectations could be an increase in the premature termination rate of apprenticeship contracts. Our state-level estimates in Table 8, however, show no evidence that changes in the BCI have a statistically significant association with changes in the rate of premature apprenticeship contract terminations in the years 2007–2018.
